# Chemometrics integrated with in silico pharmacology to reveal antioxidative and anti-inflammatory markers of dandelion for its quality control

**DOI:** 10.1186/s13020-022-00679-4

**Published:** 2022-11-04

**Authors:** Feng-Jie Liu, Jiao Yang, Xu-Yan Chen, Ting Yu, Hui Ni, Liang Feng, Ping Li, Hui-Jun Li

**Affiliations:** State Key Laboratory of Natural Medicines, China Pharmaceutical University, No. 24 Tongjia Lane, Nanjing, 210009 China

**Keywords:** Dandelion, Anti-inflammatory, Antioxidant, Chemometrics, In silico pharmacology, Quality control

## Abstract

**Background:**

Dandelion is an herb with high nutritional and medicinal values, which has been listed in Chinese Pharmacopeia, European Pharmacopoeia and British Pharmacopoeia, gaining increasing acceptance around the world. However, the current quality control of dandelion is lagging behind. Only in Chinese Pharmacopeia, cichoric acid is used as a marker compound for its quality evaluation, whereas, it can not comprehensively reflect the bioactivity of dandelion.

**Methods:**

This study developed a strategy by integrating chemometrics with in silico pharmacology to reveal the bioactive markers of dandelion for its quality control. Firstly, the major chemicals in dandelion were characterized using HPLC-DAD-MS/MS, and the corresponding antioxidant and anti-inflammatory activities were evaluated in vitro. Subsequently, the active components were screened by relating the chemicals and bioactivity of dandelion via grey relational assay and partial least squares regression analysis. The potential active components were then subjected to a validation for their activities. Moreover, in silico pharmacology was utilized to evaluate the contribution of active components to efficacy.

**Results:**

A total of 22 phenolic compounds were characterized. Among them, cichoric acid, caffeic acid and luteolin were identified as quality markers owing to their good correlations with the bioactivities of dandelion. These three markers were quantified in frequently-used dandelion species, viz. *Taraxacum mongolicum* Hand.-Mazz. (TAM) and *T. officinale* F. H. Wigg. (TAO). TAM, with acceptably higher content of cichoric acid and caffeic acid, showed better antioxidant activity than TAO. While TAO included higher content of luteolin, presenting slightly more effective in anti-inflammation.

**Conclusion:**

An useful strategy for the quality marker discovery was successfully designed. And the results provided more knowledge for the quality evaluation of dandelion.

**Supplementary Information:**

The online version contains supplementary material available at 10.1186/s13020-022-00679-4.

## Introduction

Dandelion is the entire plant of *Taraxacum mongolicum* Hand.-Mazz. (TAM), *T. officinale* F. H. Wigg. (TAO) or other sibling plants of Compositae family [1–4]. Dandelion has high nutritional and medicinal values [2, 5] and is widely used as food, nutraceuticals supplements [6], condiment [7] and for the therapy of swelling, glaucoma, hydrops, diarrhea, blisters, spleen and liver complaints [8–10]. Emerging evidences suggest dandelion contains abundant phenolic constituents with potent antioxidant, anti-inflammatory, and antimicrobial activities, resulting in diverse biological effects [2, 11, 12]. Nowadays, dandelion from different *Taraxacum* species has been listed in Chinese Pharmacopeia, European Pharmacopoeia and British Pharmacopoeia, gaining increasing acceptance around the world. However, the current quality control of dandelion is poor. Only in Chinese Pharmacopeia, cichoric acid, a high-content phenolic acid, is applied as a marker compound for its quality evaluation [13]. Whereas, there is little knowledge about whether cichoric acid can reflect the bioactivity of dandelion. Hence, the relationship of chemical constituents with the efficacy of dandelion should be explored for a deeper understanding of its quality control.

Chemometric techniques have been demonstrated to be promising tools in a wide scope of healthcare industry. It is proved that specificity may inhere between chemometric algorithms and their applied fields. Therein, grey relational assay (GRA) [14] and partial least squares regression (PLSR) [15] are the major algorithms to recognize the bioactive components from herbal products. Briefly, GRA can evaluate the similarity of variation tendency between estimate and reference variables to generate grey relational degree (GRD), which is the key parameter to reflect the correlation of each chemical component (estimate variable) with corresponding bioactivity (reference variable) [12]. PLSR, a developed generalization of multiple linear regression, can analyze data with strongly collinear, noisy and numerous X-variables, and also simultaneously model several response variables (Y-variables) [16]. PLSR provides two parameters of variable importance in the projection (VIP) and regression coefficient, which reflect the importance or correlation of each X-variables to Y. Different algorithms may lead to different results, thus, the consensus is often considered be more reliable.

On the other hand, to be effective, the molecule must reach the lesion site in sufficient concentration [17]. Similarly, as bioactive markers of an herb, the compounds should have good bioavailability. In this manner, the assessment of absorption, distribution, metabolism and excretion (ADME) of the candidate components is very necessary [18]. SwissADME is a popular web tool to in silico evaluate pharmacokinetics, drug-likeness and medicinal chemistry of small molecules based on their structures [17]. Given bioabsorption is primarily driven by gastrointestinal absorption (GA) [19], it is generally supposed that components with high GA and drug-likeness are possible to reach the site of lesion for function [20–22]. A related tool, SwissTargetPrediction could predict the targets of a given molecule, providing an economic approach to investigate its potential mechanism [23].

Herein, we integrated chemometrics with in silico pharmacology to decipher the antioxidant and anti-inflammatory markers for dandelion. At first, the phenolic constituents in dandelion were profiled using high performance liquid chromatography-diode array detection-tandem mass spectrometry (HPLC-DAD-MS/MS), and the corresponding antioxidant and anti-inflammatory activities were evaluated in vitro. Then, the active components were screened by correlating the phenolic profiles with the bioactivities of dandelion through GRA and PLSR analysis, and their capacities were validated by in vitro tests. Subsequently, in silico pharmacology was utilized to evaluate the relationship between active components and efficacy by predicting their pharmacokinetics and active targets. Finally, the efficacy-related markers were applied for the quality evaluation of dandelion from different *Taraxacum* species and geographical regions. Thus, this study provided not only a useful approach for quality marker discovery, but also further knowledge for the quality control of dandelion.

## Materials and methods

### Chemicals and reagents

Reference standards including chlorogenic acid, caftaric acid, caffeic acid, cichoric acid, luteolin, luteolin-7-*O*-*β*-d-glucoside, isochlorogenic acid A, isochlorogenic acid C, apigenin and quercetin were purchased from Chengdu Must Bio-Technology Co., Ltd. (Chengdu, China). DPPH (1,1-Diphenyl-2-picrylhydrazyl), copper (II) chloride, ascorbic acid, sodium acetate trihydrate, iron (III) chloride hexahydrate, TPTZ (2,4,6-Tris(2-pyridyl)-s-triazine), copper (III) chloride, neocuproine, Folin-Ciocalteu reagent, gallic acid, sodium nitrite, and catechin were acquired from Sigma-Aldrich Co. (St. Louis, MO, USA). All other reagents used in this study were of analytical grade.

### Samples preparation

TAM and TAO were the frequently used *Taraxacum* species of dandelion. Here, a total of 17 batches of TAM and 14 batches of TAO were collected from different regions of China. The detailed information was shown in Table 1. The plants were authenticated by an expert of State Key Laboratory of Natural Medicines, China Pharmaceutical University, and the voucher specimens (PGY-20190506) were deposited there.

The materials of dandelion were powered into homogeneous size, and sieved through a No. 60 mesh. The dried powders (0.5 g) were immersed in 20 mL of methanol and ultrasonicated for 20 min. After filtration, the supernatant was injected into HPLC-QTOF-MS for chemical characterization or lyophilized to dried powders for bioactivity analysis.

**Table 1:** The detailed information of 31 batches of dandelion

No.	Region	Origin	No.	Region	Origin
1	Hubei (HUB)	TAM	18	Sichuan (SC)	TAO
2	Hubei (HUB)	TAM	19	Gansu (GS)	TAO
3	Hubei (HUB)	TAM	20	Gansu (GS)	TAO
4	Jiangsu (JS)	TAM	21	Shanxi (SX)	TAO
5	Henan (HEN)	TAM	22	Gansu (GS)	TAO
6	Hebei (HEB)	TAM	23	Gansu (GS)	TAO
7	Jilin (JL)	TAM	24	Gansu (GS)	TAO
8	Gansu (GS)	TAM	25	Hebei (HEB)	TAO
9	Hebei (HB)	TAM	26	Jiangsu (JS)	TAO
10	Shanxi (SX)	TAM	27	Hebei (HEB)	TAO
11	Gansu (GS)	TAM	28	Hebei (HEB)	TAO
12	Gansu (GS)	TAM	29	Shanxi (SX)	TAO
13	Neimeng (NM)	TAM	30	Shanxi (SX)	TAO
14	Anhui (AH)	TAM	31	Shandong (SD)	TAO
15	Henan (HEN)	TAM			
16	Shandong (SD)	TAM			
17	Shaanxi (SAX)	TAM			

### HPLC-DAD-MS/MS analysis

The chemical profiles of dandelion were identified with Agilent 1290 HPLC system coupled with diode array detection (DAD) and Agilent 6530 QTOF-MS (Agilent Technologies, Santa Clara, CA, USA). Samples were separated on an Agilent ZORBAX Eclipse Plus C18 column (4.6 mm × 250 mm, 5 μm) held at 30 ℃. The mobile phases were 0.1% formic acid water solution (A) and methanol (B). The gradient elution conditions were set as follows: 0–7 min, 13–20% B; 7–18 min, 20–30% B; 18–28 min, 30–41% B; 28–35 min, 41–45% B; 35–38 min; 45–62% B; 38–45 min, 62–69%B; 45–50 min, 69–95% B. The flow rate and injection volume were set as 1 mL/min and 2 µL, respectively. The DAD detector scanned over the range of 190–400 nm. The electrospray ionization source was operated in the negative ionization mode. High-purity nitrogen (N_2_) was used as sheath gas (8 L/min) and nebulizing gas (35 psig). Other parameters were as follows: capillary voltage, 3500 V; collision energy, 10/20/30 eV; fragmentor voltage, 120 V; parent ion range, *m*/*z* 100–1500. Data were analyzed by MassHunter software 06.00 (Agilent Technologies, Santa Clara, CA, USA).

### HPLC-UV analysis

The quantitative analysis of identified compounds was performed by Shimadzu LC-20AT HPLC system (Shimadzu, Kyoto, Japan.) coupled with G4212B DAD. An Agilent ZORBAX Eclipse Plus C18 column (4.6 mm × 250 mm, 5 μm) was used for chromatographic separation. The mobile phases and elution program were the same as those described in Section "HPLC-DAD-MS/MS analysis". The injection volume was set as 10 µL and the data were acquired at the wavelength of 327 nm.

### Antioxidant activity

#### Ferric ion reducing antioxidant power (FRAP) assay

The FRAP assay was performed as previously described [24]. FRAP reagent was freshly prepared by mixing together 10 mM 2,4,6-tripyridyl-s-triazine (TPTZ) and 20 mM ferric chloride in 0.25 M acetate buffer, pH 3.6. dandelion sample (100 µL) was added to 300 µL of water followed by 3 mL of FRAP reagent at 1 min intervals. The absorbance was read at 593 nm after 4 min incubation at ambient temperature against distilled water. A calibration curve of ferrous sulfate (100–1000 µmol/L) was used, and results were expressed in µmol Fe (II)/g dandelion and in mmo1/L for reference standards from three determinations.

#### DPPH radical scavenging assay

The method was performed based on previous literature [25] with some modifications. Briefly, 20 µL of sample solution and 180 µL of DPPH (0.2 mM dissolved in methanol) were added to a 96-well microplate and incubated at 37 ℃ in dark for 30 min. The absorbance of the reaction at 517 nm was recorded with a microplate reader (Thermo scientific, Shanghai, China). Trolox and 80% methanol were used as positive and negative control, respectively. DPPH radical scavenging activity was calculated according to the following equation:

$$DPPH\,scavenging\,capacity\left(\%\right)=\left(1-\frac{{A}_{2}-{A}_{0}}{{A}_{1}}\right)$$

where A_0_ was the absorbance of the blank group (without DPPH); A_1_ was the absorbance of negative control group; A_2_ was the absorbance of the test sample group.

### Evaluation of anti-inflammatory activity

The anti-inflammatory activity of the extracts was evaluated based on the nitric oxide (NO) production by lipopolysaccharide (LPS)-stimulated RAW 264.7 cells. RAW 264.7 cells were cultured in DMEM medium supplemented with 10% heat-inactivated fetal bovine serum (FBS), 100 U/ml of penicillin and 100 µg/ml of streptomycin and maintained at 37 °C in a 5% CO_2_ humidified atmosphere (CO_2_ incubator, Heal Force). RAW264.7 cells were pretreated with dandelion samples (12.5, 25, 50, 100, 200, 400 µg/mL) for 4 h, and then stimulated with or without LPS (2 µg/mL) for 24 h. The supernatants were collected to determine NO production by Griess reagent (Beyotime Biotechnology, China). The residues were added with CCK-8 reagent to evaluate the cell viability. The results were expressed as IC_50_ values (µg/mL), which corresponded to the dandelion concentration that induced 50% of inhibition of NO production.

### Chemical-efficacy relationship

#### Grey relational analysis

Antioxidant and anti-inflammatory activities of 31 samples were selected as the reference variables and the peak areas of 22 phenolic components were defined as estimate variables. To assess the chemical-efficacy relationship, GRD between phenolic components and bioactivities was calculated by Scientific Platform Serving for Statistics Professional (SPSSPRO, https://www.spsspro.com/).

#### Partial least squares regression

The correlations between the relative contents of 22 phenolic peaks (predictor variables X) and the antioxidant and anti-inflammatory activities (response variables Y), respectively were modeled by PLS using SIMCA version 14.0.1 (Umetrics AB, Umea, Sweden). Two model parameters, Q^2^ (> 0.5 indicates good predictivity) and correlation coefficient (R^2^Y), were used for estimation. The peaks with variable importance in the projection (VIP) > 1 and regression coefficient > 0.1 were selected as the candidate markers.

### In silico pharmacology

The Canonical SMILES of each candidate marker was imported to the web tool of SwissADME (http://www.swissadme.ch/) to calculate its pharmacokinetic parameters and to SwissTargetPrediction (http://www.swisstargetprediction.ch/) to predict its targets. The functions of the targets were annotated and enriched by the database for annotation, visualization and integrated discovery (DAVID, https://david.ncifcrf.gov/home.jsp). Compounds with good GA and drug-likeness were considered to be accessible to the body for function. And the target functional and enrichment analysis could reveal the potential mechanisms of efficacy-related markers.

## Results and discussions

### Identification of phenolic constituents in dandelion

**Fig. 1:**
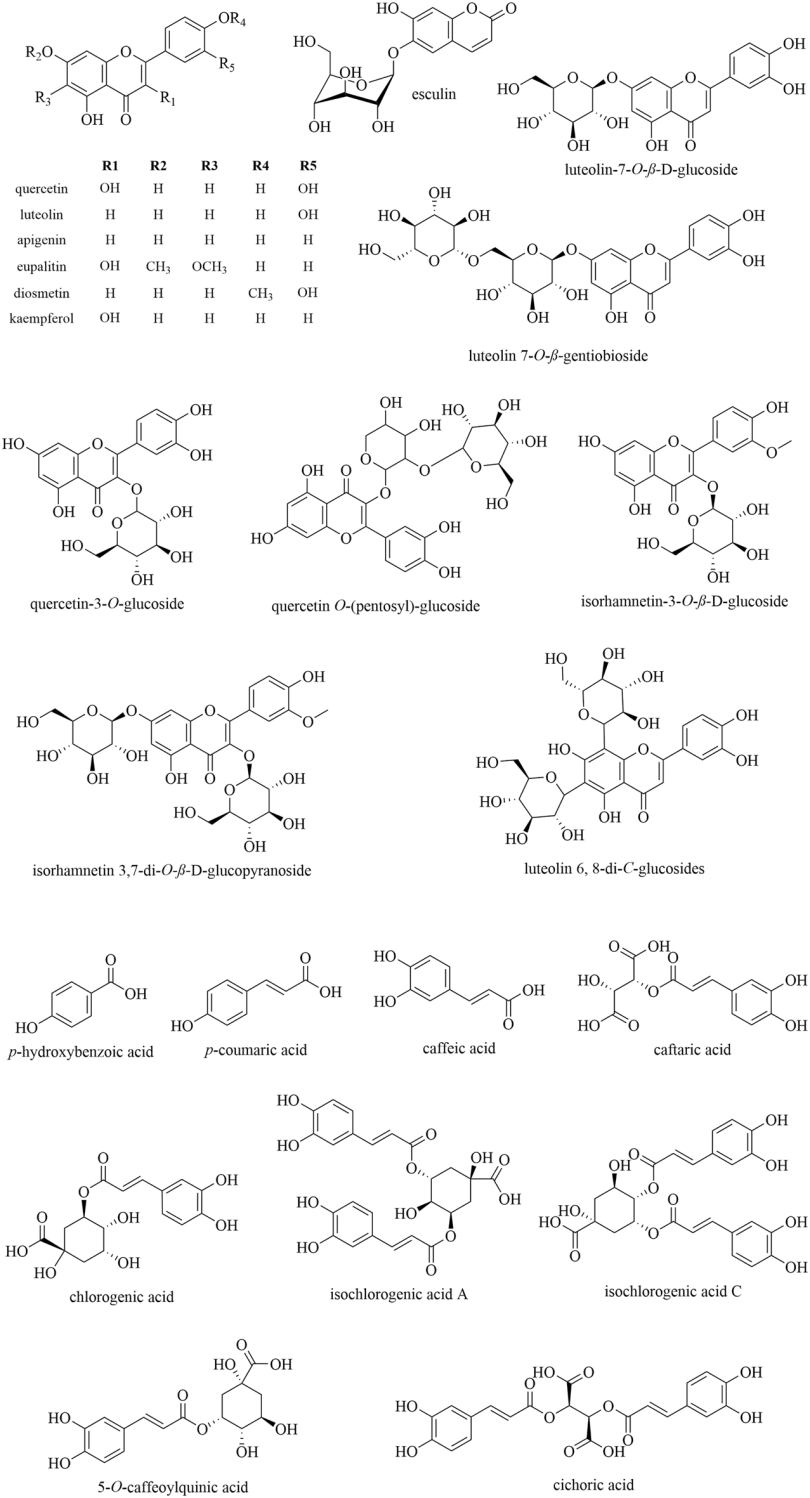
Chemical structures of 22 phenolic compounds in dandelion

HPLC-DAD-MS/MS was applied to analyze phenolic components in dandelion. Due to the special conjugated system, phenolic compounds have characteristic UV absorbance spectra [26]. Such a property is a core clue to recognize phenolic compounds from others. Together with MS/MS spectra, a total of 22 phenolic components were tentatively identified, including 9 phenylpropionic acids and 13 flavonoids (Fig. 1). Representative UV absorbance spectra and base peak chromatograms are shown in Additional file 1: Fig. S1. Detailed information on the identified peaks, including the retention time, molecular formula, ion type, detected *m/z*, and fragment ions, are summarized in Additional file 1: Table S1.

#### Identification of phenylpropionic acids and their derivatives

**Fig. 2:**
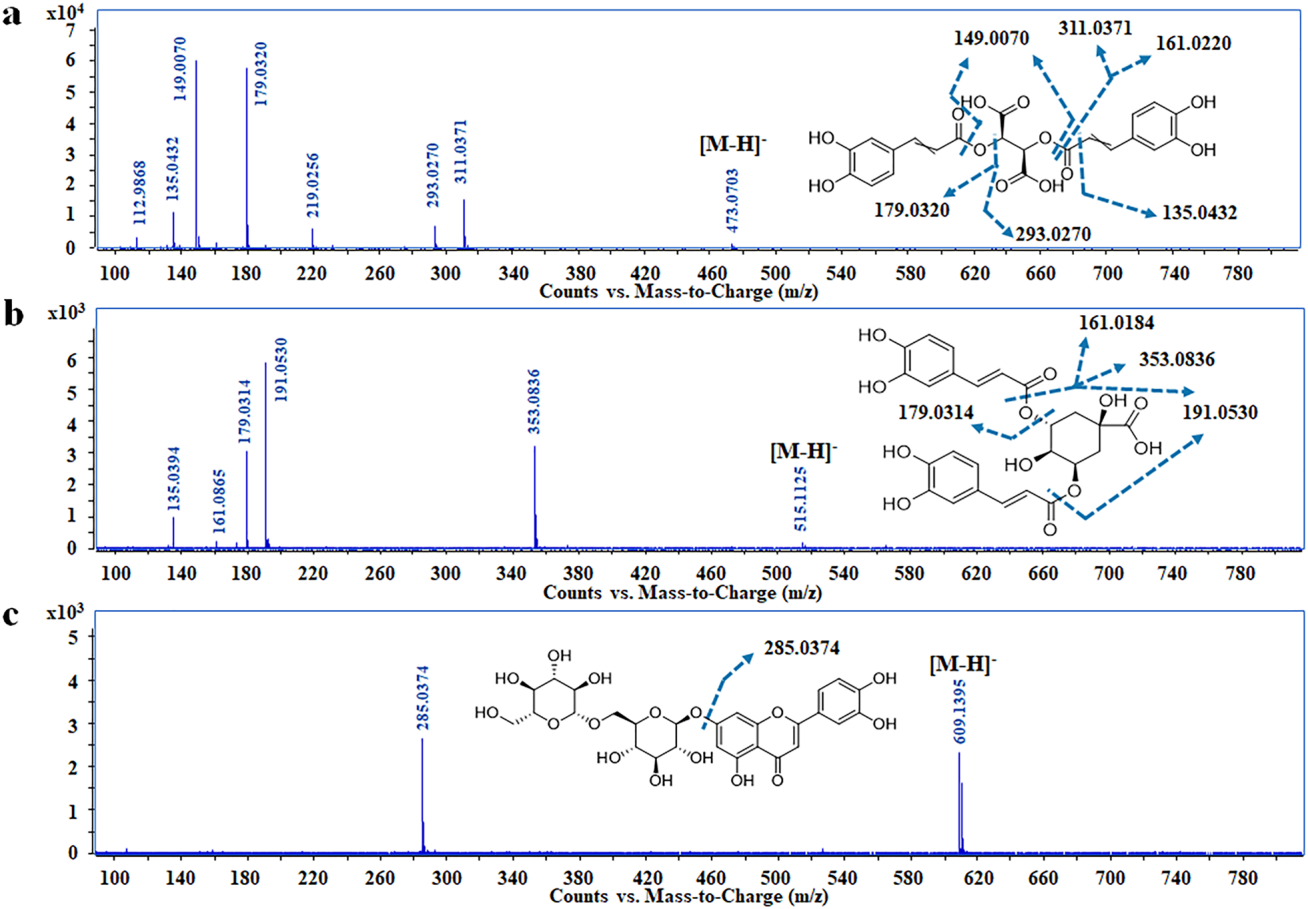
The proposed fragmentation pathway of representative compounds in the negative MS/MS spectrum. **a** peak **7** (cichoric acid), **b** peak **11** (isochlorogenic acid A), **c** peak **9** (luteolin-7-*O*-*β*-d-gentiobioside)

Among the nine phenylpropionic acids, seven of them were positively identified as caffeic acid (peak **5**), caffeoyltartaric acids (peaks **1** and **7**) and caffeoylquinic acids (peaks **3**, **4**, **11** and **14**) by comparing with reference standards. These compounds were generalized by quinic or tartaric acid with several caffeic acids, thus, the predominant fragment ions were produced by the successive loss of caffeic acid and tartaric acid/quinic acid moieties. For example, cichoric acid (peak **7**) could lose caffeic acid moiety to generate the fragment ion of *m/z* 293.0270, and then lose tartaric acid to produce *m/z* 179.0320; isochlorogenic acid A (peak **11**) produced the fragment ions at *m/z* 353.0836 and *m/z* 191.0526 by the stepwise loss of caffeic acid and quinic acid moieties (Fig. 2a). In addition, peak **2**, showing a precursor ion of *m/z* 137.0220 and fragment ions of *m/z* 119.0122, *m/z* 108.0208 and *m/z* 92.0255, was putatively identified as *p*-hydroxybenzoic acid according to the literature [27]. Similarly, Peaks **8** was putatively identified as *p*-coumaric acid [28].

#### Identification of flavonoids

Flavonoids in dandelion had a strong UV absorbance band between 300 and 390 nm, indicating they involved cinnamoyl structure and belonged to flavone or flavonol [26]. Given the MS/MS fragmentation, flavonoids could generate deprotonated ions [M-H]^−^ in negative ion mode and the prominent fragments were produced through C-ring (central three-carbon chain) cleavage, successive losses of substituents and/or glycose moieties. Among the 13 detected flavonoids, five were positively identified as luteolin (peak **18**), apigenin (peak **20**), luteolin-7-*O*-*β*-d-glucoside (peak **12**), quercetin (peak **17**) and kaempferol (peak **19**) by comparing with reference standards. The other 8 compounds were tentatively annotated as the glycosides or derivatives of luteolin, kaemplerol and apigenin. Take peak **9** for an example, it exhibited an [M-H]^−^ ion at *m/z* 609.1461 and generated a base peak at *m/z* 285.0374 (Fig. 2c), indicating that peak **9** was a derivative of luteolin. The neutral loss of 324.0987 (C_12_H_20_O_10_) corresponded to a substitute of disaccharide, thus, it was putatively identified as luteolin-7-*O*-*β*-d-gentiobioside (Fig. 2c) [29].

### Antioxidant activity of dandelion

**Fig. 3:**
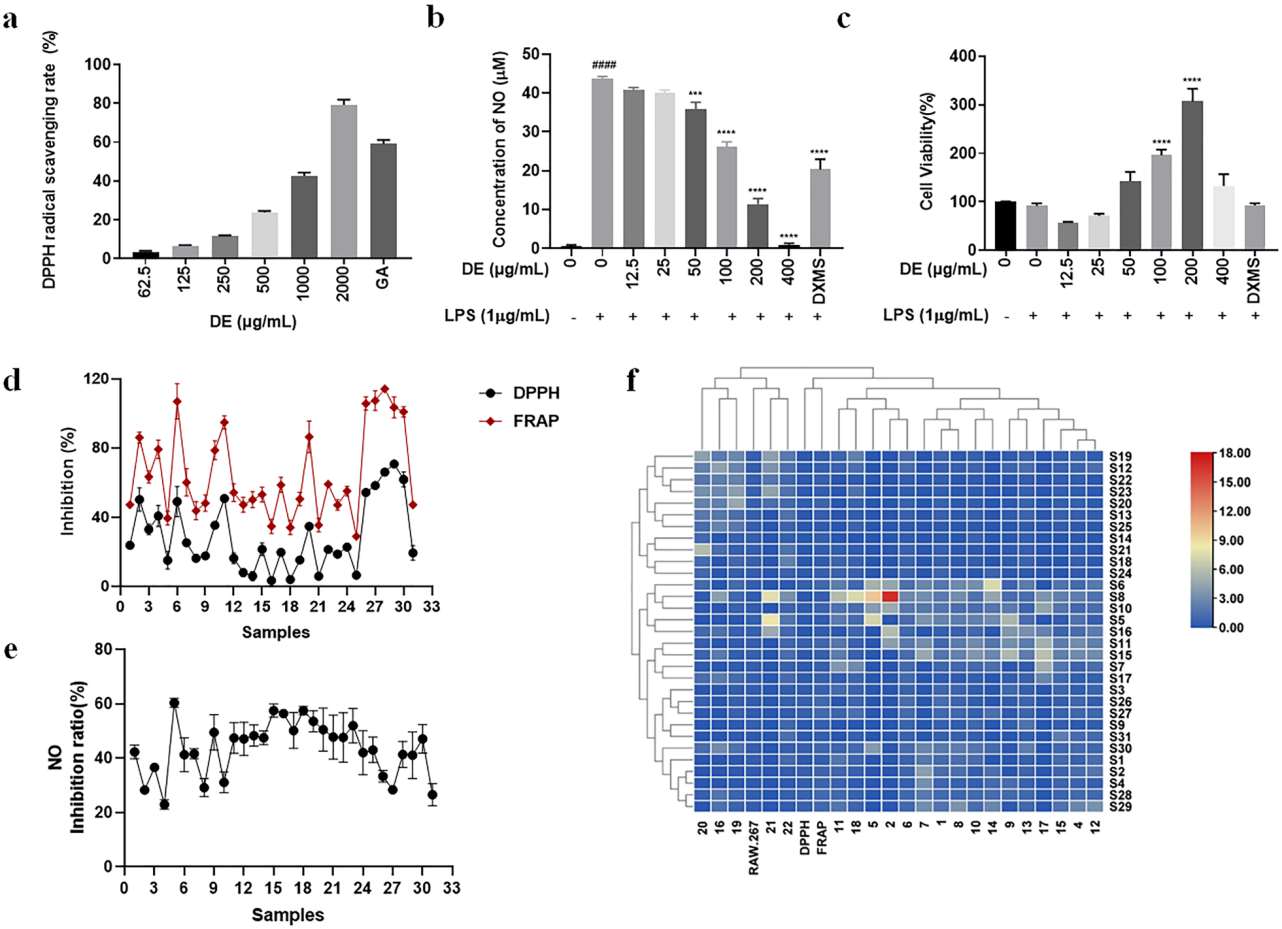
The bioactivity and chemical profile of dandelion. **a** the antioxidant activity represented by radical scavenging rates of dandelion at different concentrations and gallic acid (GA, 1.6 µg/mL); **b** the anti-inflammatory activity represented by LPS-induced NO production of dandelion and dexamethasone (DXMS, 2 µg/mL) and **c** their cytotoxicity on RAW 264.7 cells at different concentrations; **d** antioxidant activity of different dandelion samples at 500 µg/mL; **e** anti-inflammatory activity of different dandelion samples at 100 µg/mL; **f** the correlation heatmap of chemicals and active capacity of dandelion samples. DE: dandelion extract. Data is expressed as mean ± SEM of three experiments (n = 3) performed in triplicate, one-way ANOVA, ^####^p < 0.0001, LPS (1 µg/mL) vs. control group; ***p < 0.001, ****p < 0.0001, other groups vs. LPS (1 µg/mL)

Two approaches of DPPH and FRAP were applied to evaluate the antioxidant activity of dandelion, which was represented by the of radical scavenging (DPPH) or electron transfer rate (FRAP). Gallic acid with good antioxidant activity was used as positive control drug. As shown in Fig. 3a, dandelion scavenged radicals in a concentration-dependent manner in the range of 62.5–2000 µg/mL, which was a prerequisite to unclose active components through chemical-efficacy relationship. Then the concentration of 500 µg/mL was chosen for the activity evaluation of 31 batches of dandelion. The antioxidative capacity ranged from 1.9 to 71.05% and 28.99–114.10% based on DPPH and FRAP, respectively (Fig. 3d).

### Anti-inflammatory activity of dandelion

At first, the cytotoxicity of dandelion sample on RAW 264.7 cells was measured by CCK-8 assay. RAW 264.7 cells treated with dexamethasone were utilized as positive control. As shown in Fig. 3c, the dandelion had no toxicity to RAW 264.7 cells in the range of 12.5–400 µg/mL and significantly promoted cell proliferation in the range of 100–200 µg/mL. The secretion of NO in RAW 264.7 cells was extremely low (0.67 µM) without stimulus, while LPS induced a significant increase of NO level (43.72 µM). When treated with a series of concentration of dandelion (12.5–400 µg/mL), the secretion of NO was dose-dependently reversed and the inhibition rate varied over the range of 6.02–97.83% (Fig. 3b). Then the concentration of 100 µg/mL was applied to evaluate the anti-inflammatory capability of 31 batches of dandelion, which ranged from 22.99 to 60.36% (Fig. 3e). Furthermore, Fig. 3f showed diverse Pearson distances among the chemicals and antioxidant/anti-inflammatory activities of dandelion, which indicated that the potential active components could be discovered by chemicals-efficacy relationship.

### Correlations between the phenolic components and bioactivities

GRA was primarily used to assess the correlation of each phenolic component annotated in dandelion with antioxidant and anti-inflammatory activities. The GRD values of 22 phenolic compounds were over 0.9 (Table 2), indicating their high correlation with corresponding activities. The results agreed with the previous view that phenolic compounds are the major components responsible for antioxidant and anti-inflammatory activities [30, 31].

**Table 2:** The grey relational grade of phytochemical components and bioactivities

No.	DPPH	FRAP	Anti-inflammatory	No.	DPPH	FRAP	Anti-inflammatory
**1**	0.971	0.974	0.966	**12**	0.961	0.966	0.968
**2**	0.911	0.910	0.908	**13**	0.948	0.944	0.931
**3**	0.964	0.967	0.961	**14**	0.959	0.962	0.956
**4**	0.914	0.910	0.908	**15**	0.919	0.925	0.932
**5**	0.967	0.974	0.972	**16**	0.911	0.910	0.913
**6**	0.936	0.926	0.917	**17**	0.948	0.955	0.961
**7**	0.963	0.957	0.951	**18**	0.935	0.945	0.953
**8**	0.939	0.939	0.939	**19**	0.924	0.934	0.948
**9**	0.957	0.959	0.960	**20**	0.904	0.906	0.914
**10**	0.951	0.949	0.948	**21**	0.914	0.914	0.917
**11**	0.963	0.961	0.955	**22**	0.940	0.950	0.971

### Screening of antioxidant and anti-inflammatory candidate markers of dandelion

PLSR was utilized to construct the relationship between 22 phenolic compounds and corresponding activities for the screening of efficacy-related markers.

For antioxidant capacity, the parameters of R^2^Y (cum) & Q^2^ (cum) in the PLSR models about DPPH and FRAP were 0.904 & 0.753 and 0.861 & 0.656, respectively, indicating a satisfactory explanation and prediction ability. Permutation tests verified the models not overfitted (Additional file 1: Fig. S2). Following the application of PLSR models, the regression coefficients were obtained, which could reflect the positive or negative contribution of each peak to the activity. And higher coefficient values implied the variables more important for the activity. VIP value was another indispensable parameter that was usually employed to screen the important variables contributing to the bioactivity. Overall, variables with coefficient > 0.1 & VIP > 1.0 were signed as candidate bioactive compounds. Due to the different sensitivities, the important components screened by DPPH or FRAP were combined to reduce the false negative results. As shown in Table 3, peaks **1**, **3**, **5**, **7** and **18** were identified to be the potential components responsible for antioxidant activity of dandelion.

**Table 3:** The potential antioxidant constituents of dandelion

No.	DPPH		FRAP		Compound
	VIP	Coefficient	VIP	Coefficient	
**1**	1.52	0.40	1.45	0.35	Caftaric acid
**3**	1.15	0.29	1.05	0.26	5-*O*-caffeoylquinic acid
**5**	1.03	0.28	1.28	0.43	Caffeic acid
**7**	1.39	0.19	1.28	0.12	Cichoric acid
**18**	0.89	0.24	1.01	0.31	Luteolin

Similarly, the PLSR model about anti-inflammatory activity was also constructed in Additional file 1: Fig. S2 (R^2^Y (cum), 0.619 & Q^2^ (cum), 0.507). The same standards were applied to screen out the potential anti-inflammatory components. As shown in Table 4, peaks **8**, **9**, **13**, **17**, **18, 19**, **20** and **21** were considered to be responsible for the anti-inflammatory activity of dandelion.

**Table 4:** The potential anti-inflammatory constituents of dandelion

No.	VIP	Coefficient	Compound
**8**	1.32	0.13	*p*-Coumaric acid
**9**	1.28	0.12	Luteolin-7-*O*-*β*-d-gentiobioside
**13**	1.31	0.13	Luteolin-7-*O*-*β*-d-glucoside
**17**	1.01	0.11	Isorhamnetin-3-*O*-*β*-d-glucoside
**18**	1.07	0.13	Luteolin
**19**	1.82	0.21	Kaempferol
**20**	1.31	0.16	Apigenin
**21**	1.53	0.17	Eupalitin

### Activity evaluations of candidate markers in vitro

#### Evaluation of antioxidant activity

**Fig. 4:**
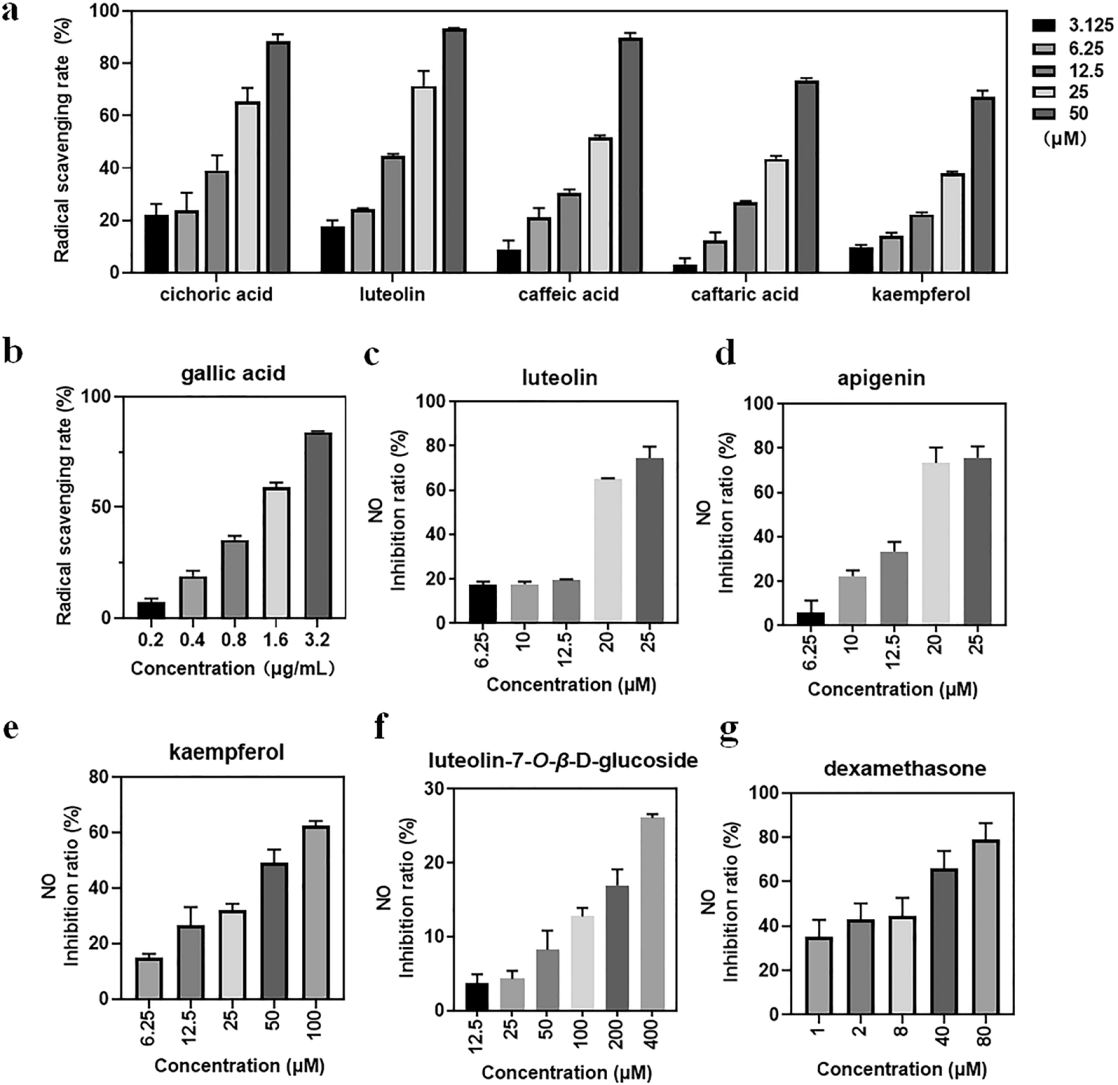
The bioactivity evaluation of the potential active compounds. The antioxidant capacity of five potential compounds (**a**) and gallic acid (**b**); the anti-inflammatory capacity of four candidate compounds (**c–f**) and dexamethasone (**g**)

As shown in Fig. 4 and Additional file 1: Table S2, luteolin, cichoric acid, 5-*O*-caffeoylquinic acid, caffeic acid and caftaric acid presented good antioxidant activity and their IC_50_ values were 13.15 ± 0.52 µM, 14.17 ± 2.50 µM, 36.76 ± 0.85 µM, 19.63 ± 0.60 µM and 26.53 ± 0.40 µM, respectively. Take Trolox (or FeSO_4_) as a reference, the antioxidant capacities of the four compounds were signed as 6.46 ± 1.49 mmol Trolox/g (11.01 ± 1.49 mmol FeSO_4_/g), 3.93 ± 0.49 mmol Trolox/g (9.39 ± 0.87 mmol FeSO_4_/g), 2.54 ± 0.64 mmol Trolox/g (4.66 ± 1.20 mmol FeSO_4_/g), 8.25 ± 0.78 mmol Trolox/g (18.70 ± 3.96 mmol FeSO_4_/g)) and 3.94 ± 0.30 mmol Trolox/g (10.46 ± 1.27 mmol FeSO_4_/g).

#### Evaluation of anti-inflammatory activity

As shown in Table 4, flavonoids were the major constituents that were responsible for the anti-inflammatory activity of dandelion. These constituents possessed the same flavone skeleton with diverse substituents at positions of C3, C6, C7 and C3′ (Fig. 1). To investigate the structure-efficacy relationship of these flavones, four typical compounds with available standards were included to evaluate their anti-inflammatory activity. As shown in Fig. 4, luteolin performed excellent anti-inflammatory activity (IC_50_, 17.70 µM), which was comparable to that of apigenin (IC_50_, 15.61 µM) but far superior to kaempferol (IC_50_, 54.17 µM) and luteolin-7-*O*-*β*-d-glucoside (IC_50_, > 400 µM). Comparing their structural characteristics, it could be deduced that the chromonyl group was the anti-inflammatory pharmacophore.

Additionally, the cytotoxicity of the anti-inflammatory compounds was investigated (Additional file 1: Fig. S3). The four flavones showed no significant toxicity to RAW264.7 cells in the range of 6.25–25 µM. When the concentration was over 25 µM, kaempferol and luteolin-7-*O*-*β*-d-glucoside presented a mild dose-dependent toxicity.

### In silico pharmacology evaluation of candidate quality markers

To be effective markers, the molecules should reach their targets in the body in sufficient concentration. SwissADME (http://www.swissadme.ch/), was applied to predict the pharmacokinetics parameters (GA and drug-likeness) of these candidate markers. Drug-likeness was evaluated by Lipinski, Ghose, Veber, Ega, and Muegge rules, which were developed by major pharmaceutical companies based on the structural or physicochemical inspections to exclude molecules with properties most probable incompatible with an acceptable pharmacokinetics profile [17]. Generally, when GA was “high” and over two parameters of drug-likeness were “yes”, the compounds were considered to be accessible to the body for functions. As shown in Additional file 1: Table S3, only peaks **18**, **20**, **19**, **5**, **8** and **21** conformed to the above conditions. Thus, they were theoretically reasonable to be markers for the quality control of dandelion. Moreover, although the GA of peak **7** was low, it could be detected in vivo [32] due to its high content (> 20 times more than the other constituents). Thus, peak **7** was exceptionally involved in the quality markers.

**Fig. 5:**
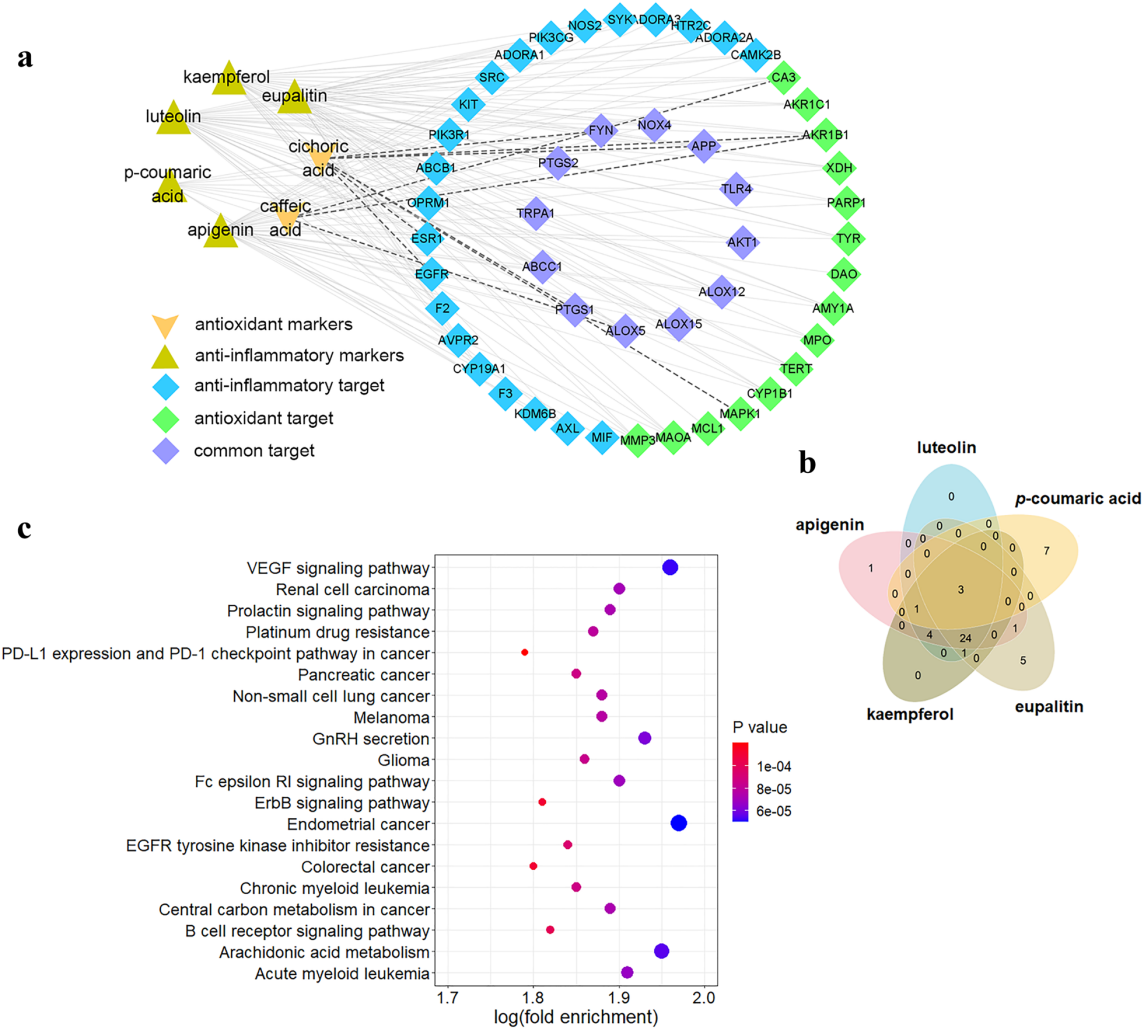
In silico pharmacology. **a** the antioxidant and anti-inflammatory targets of candidate markers predicted by SwissTargetPrediction, **b** Venn plot of flavones with highly overlapped targets, and **c** KEGG pathway enrichment analysis based on the predicted targets

The targets of the above 7 markers (peak **18**, **20**, **19**, **5**, **8**, **21**, and **7**) were predicted based on their structures using SwissTargetPrediction web tool (http://www.swisstargetprediction.ch/). The obtained targets were annotated *via* DAVID and then a total of 49 targets related to oxidation and inflammation were discovered. The results were visualized in Fig. 5a. The antioxidative markers of caffeic acid (peak **5**) and cichoric acid (peak **7**) targeted ALOX5, CA3, FYN, MAPK1, APP, PTGS1 and AKR1B1, while the 5 anti-inflammatory markers targeted up to 47 unique genes. As shown in Fig. 5b, the anti-inflammatory markers had high overlapped targets: approximately 70% of the 47 unique genes were targeted by at least 3 markers, which could attribute to their similar structures. These genes mainly functioned in the biological processes of innate/adaptive immunity, inflammatory response, lipid and glucose metabolism, host-virus interaction, and apoptosis, which supported the claimed efficacy of dandelion such as swelling and pain of eye, mammary/pulmonary abscess, scrofula, and urinary infection [2, 7]. The top 20 pathways of KEGG functional enrichment analysis (Fig. 5c) included kinds of carcinomas, VEGF signaling pathway, platinum drug resistance, immune checkpoint pathway, B cell receptor signaling pathway and central carbon metabolism of cancer, etc. indicating the promising potential of dandelion for the treatment of cancers [32–34].

### Application of marker compounds in the quality control of dandelion

As shown in Additional file 1: Fig. S4, 80% methanol, resulting in a better extraction efficiency of phenolic components, was used to prepare samples for quality control. The contents of *p*-coumaric acid, eupalitin, kaempferol and apigenin always fluctuated around the limit of quantitation, indicating their unsatisfying measurability. Given the excellent bioactivity (Fig. 4) and high overlapped targets (Fig. 5b), it was supposed that luteolin could represent the bioactivity of flavones in dandelion. Therefore, cichoric acid, caffeic acid and luteolin were considered as markers for the quality control of dandelion.

**Fig. 6:**
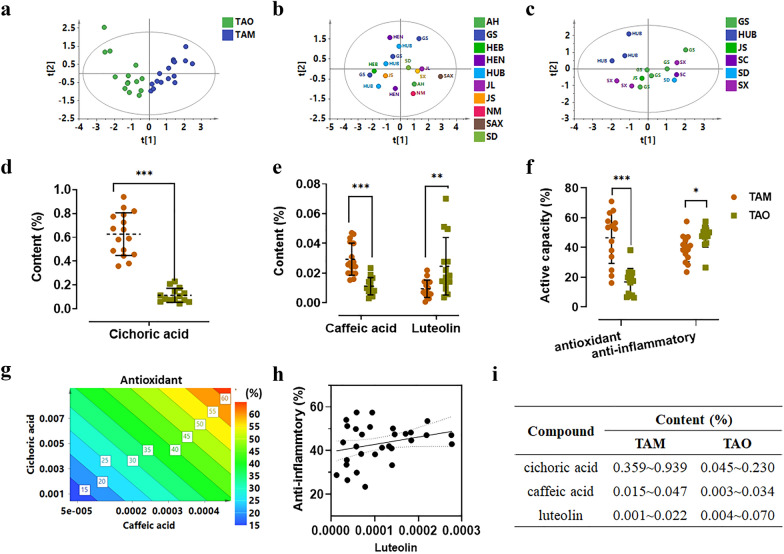
The application for quality control of dandelion. **a** PCA score plot of TAM&TAO, **b** PCA score plot of TAM, **c** PCA score plot of TAO, **d**, **e** distribution of quality markers and **f** active capacity of dandelion from different species, **g** the response of cichoric acid and caffeic acid to antioxidant activity, **h** the response of luteolin to anti-inflammatory activity, **i** content of quality markers in dandelion

A simultaneous quantitation method was developed based on HPLC-UV. Methodology investigation was performed (Additional file 1: Table S4), showing good linearity with correlation coefficients (r^2^) higher than 0.9994 in a in a relatively wide concentration range. The contents of three markers in the frequently used dandelion species of TAM and TAO were determined. As shown in Fig. 6a, PCA analysis could obviously distinguish TAM from TAO. The content of cichoric acid and caffeic acid in TAM was significantly higher than that in TAO (Fig. 6d and e). Meanwhile, TAM showed much higher antioxidant activity than TAO (Fig. 6f), further demonstrating that cichoric acid and caffeic acid could be the antioxidant markers of dandelion. That was also supported by the activity-response spectrum of cichoric acid and caffeic acid (Fig. 6g). On the other hand, despite no statistic difference, the content of luteolin and anti-inflammatory activity showed similar tendency between TAM and TAO (Fig. 6e, f and h). Focusing on the effects of geographic regions, the samples belonging to the same species were analyzed. As shown in Fig. 6b and c, the samples from different regions dispersed in the PCA score plots, indicating the quality of dandelion was not susceptible to regions.

## Conclusion

In this study, a strategy of integrating chemometrics with *in silico* pharmacology was developed to decipher active markers of dandelion for its quality control. A total of 22 phenolic components were characterized in dandelion, including 9 phenylpropionic acids and 13 flavonoids. Among them, cichoric acid, caffeic acid and luteolin with good antioxidant/anti-inflammatory activities were identified as the quality markers of dandelion. For quality evaluation, the three compounds were quantified in the frequently used dandelion species of TAM and TAO. Therein, TAM contained significantly higher cichoric acid and caffeic acid, showing better antioxidant activity than TAO. While TAO included higher content of luteolin, presenting a slight advantage on anti-inflammatory effect. These results provide further knowledge for the quality control of dandelion, and the strategy could be extended to other herbal medicines.

## Supplementary Information

**Additional file 1**: **Table S1.** The identification of phenolic components in TH.**Table S2. **Antioxidant capacity of candidate markers. **Table S3.** The in silico pharmacokinetic parametersof candidate markers. **Table S4. **Methodology investigation for thequantitation of four markers. **Figure S1.** Representative UV absorbanceprofile (**a**), and base peak chromatogram (**b**). **Figure S2. **PLSRmodels. The summary of fit (**a-c**) and permutation tests (**d-f**) forantioxidative activity by DPPH and FRAP, and anti-inflammatory activity,respectively. **Figure S3. **The toxicity of luteolin (**a**), apigenin (**b**),kaempferol (**c**) and luteolin-7-*O*-*β*-d-glucoside (**d**) on RAW264.7 cells. **Figure S4. **Chromatogramsof *Taraxacum* extracts with different extraction solvents. **1**caffeic acid, **2** cichoric acid, **3** p-coumaric acid, **4**luteolin, **5** kaemferol, **6** apigenin, **7** eupalitin.

## Data Availability

All data generated or analysed during this study are included in this published article and its supplementary information files.
