# Supplementary material for: Chemometrics integrated with in silico pharmacology to reveal antioxidative and anti-inflammatory markers of dandelion for its quality control
Source: Chin Med. 2022 Nov 4;17:125. doi: 10.1186/s13020-022-00679-4 (PMC9636813; doi:10.1186/s13020-022-00679-4)
Supplement: Supplementary file 1 — Additional file 1: Table S1. The identification of phenolic components in TH.Table S2. Antioxidant capacity of candidate markers. Table S3. The in silico pharmacokinetic parametersof candidate markers. Table S4. Methodology investigation for thequantitation of four markers. Figure S1. Representative UV absorbanceprofile (a), and base peak chromatogram (b). Figure S2. PLSRmodels. The summary of fit (a-c) and permutation tests (d-f) forantioxidative activity by DPPH and FRAP, and anti-inflammatory activity,respectively. Figure S3. The toxicity of luteolin (a), apigenin (b),kaempferol (c) and luteolin-7-O-β-d-glucoside (d) on RAW264.7 cells. Figure S4. Chromatogramsof Taraxacum extracts with different extraction solvents. 1caffeic acid, 2 cichoric acid, 3 p-coumaric acid, 4luteolin, 5 kaemferol, 6 apigenin, 7 eupalitin. [file 13020_2022_679_MOESM1_ESM.docx]

Additional file 1

**Chemometrics integrated with *in silico* pharmacology to reveal antioxidative and anti-inflammatory markers of dandelion for its quality control**

Feng-Jie Liu^1*^, Jiao Yang^1^, Xu-Yan Chen, Ting Yu, Hui Ni, Liang Feng, Ping Li, Hui-Jun Li^*^

State Key Laboratory of Natural Medicines, China Pharmaceutical University, No. 24 Tongjia Lane, Nanjing 210009, China

^*^ Corresponding authors:

E-mail addresses: liufj_cpu@126.com (Feng-Jie Liu); cpuli@163.com (Huijun Li).

^1^ The authors contributed equally to this work.

**Contents**

**Table S1** The identification of phenolic components in TH.

**Table S2** Antioxidant capacity of candidate markers

**Table S3** The *in silico* pharmacokinetic parameters of candidate markers.

**Table S4** Methodology investigation for the quantitation of four markers.

**Fig. S1** Representative UV absorbance profile (**a**), and base peak chromatogram (**b**).

**Fig. S2** PLSR models. The summary of fit (**a-c**) and permutation tests (**d-f**) for antioxidative activity by DPPH and FRAP, and anti-inflammatory activity, respectively.

**Fig. S3** The toxicity of luteolin (**a**), apigenin (**b**), kaempferol (**c**) and luteolin-7-*O*-*β*-d-glucoside (**d**) on RAW264.7 cells.

**Fig. S4** Chromatograms of *Taraxacum* extracts with different extraction solvents. **1** caffeic acid, **2** cichoric acid, **3** p-coumaric acid, **4** luteolin, **5** kaemferol, **6** apigenin, **7** eupalitin.

**Table S1** The identification of phenolic components in TH.

| No. | Rt/min | *m/z* | Ion type | Formula | MS/MS fragments | Identification |
| --- | --- | --- | --- | --- | --- | --- |
| 1 | 10.945 | 311.0324 | [M-H]^-^ | C_13_H_12_O_9_ | 179.0320/149.0069/135.0426/133.0221 | caftaric acid |
| 2 | 13.089 | 137.0220 | [M-H]^-^ | C_7_H_6_O_3_ | 119.0122/108.0208/92.0255 | *p*-hydroxybenzoic acid |
| 3 | 16.306 | 353.0817 | [M-H]^-^ | C_16_H_18_O_9_ | 191.0534/179.0331 | 5-*O*-caffeoylquinic acid |
| 4 | 16.34 | 353.0533 | [M-H]^-^ | C_16_H_18_O_9_ | 191.0526/173.0424/85.0285 | chlorogenic acid |
| 5 | 19.523 | 179.0318 | [M-H]^-^ | C_9_H_8_O_4_ | 135.0429/89.0397 | caffeic acid |
| 6 | 22.231 | 595.1248 | [M-H]^-^ | C_26_H_28_O_16_ | 463.0833/301.0301 | quercetin *O*-(pentosyl)-glucoside |
| 7 | 25.153 | 473.0703 | [M-H]^-^ | C_22_H_18_O_12_ | 311.0371/149.0070/219.0256/293.0270/179.0320 | cichoric acid |
| 8 | 25.957 | 163.0376 | [M-H]^-^ | C_9_H_8_O_3_ | 119.05 | *p*-coumaric acid |
| 9 | 29.04 | 609.1395 | [M-H]^-^ | C_27_H_30_O_16_ | 285.0374/327.0466 | luteolin-7-*O*-*β*-d-gentiobioside |
| 10 | 29.358 | 611.1574 | [M+H]^+^ | C_27_H_30_O_16_ | 497.0992/449.1050/365.1020/287.0537 | luteolin-6, 8-di-*C*-glucosides |
| 11 | 31.453 | 515.1125 | [M-H]^-^ | C_25_H_24_O_12_ | 353.0836/191.0530/161.0865 | isochlorogenic acid A |
| 12 | 32.66 | 447.0865 | [M-H]^-^ | C_21_H_20_O_11_ | 285.0358 | luteolin-7-*O*-*β*- d -glucoside |
| 13 | 33.648 | 463.0848 | [M-H]^-^ | C_21_H_20_O_12_ | 301.0297/273.0413/178.9958/151.0014 | quercetin-3-*O*-glucoside |
| 14 | 36.011 | 515.1178 | [M-H]^-^ | C_25_H_24_O_12_ | 353.0820/191.0613 | isochlorogenic acid C |
| 15 | 38.44 | 639.3069 | [M-H]^-^ | C_28_H_32_O_17_ | 519.2529/477.2481/373.2224/145.0272/119.0503 | isorhamnetin-3,7-di-*O*-*β*-d-glucoside |
| 16 | 38.741 | 477.1060 | [M-H]^-^ | C_22_H_22_O_12_ | 314.04 | isorhamnetin-3-*O-β*-d-glucoside |
| 17 | 40.568 | 301.0308 | [M-H]^-^ | C_15_H_10_O_7_ | 178.9958/151.0014/121.0280 | quercetin |
| 18 | 41.389 | 285.0364 | [M-H]^-^ | C_15_H_10_O_6_ | 151.0012/133.0278 | luteolin |
| 19 | 42.086 | 285.0411 | [M-H]^-^ | C_15_H_10_O_6_ | 151.0000/133.0270/65.0038 | kaempferol |
| 20 | 42.445 | 269.0408 | [M-H]^-^ | C_15_H_10_O_5_ | 225.0492/117.0324/151.0008/65.0032 | apigenin |
| 21 | 42.638 | 329.0632 | [M-H]^-^ | C_17_H_14_O_7_ | 314.04 | eupalitin |
| 22 | 42.723 | 299.0530 | [M-H]^-^ | C_16_H_12_O_6_ | 284.0286/256.0343 | diosmetin |

**Table S2** Antioxidant capacity of candidate markers

| Compound | IC_50_ DPPH  (μM） | DPPH  (mmol Trolox/g) | FRAP  (mmol FeSO_4_/g) |
| --- | --- | --- | --- |
| luteolin | 13.15±0.52 | 6.46±1.49 | 11.01±2.99 |
| cichoric acid | 14.77±2.50 | 3.93±0.49 | 9.39±0.87 |
| caffeic acid | 19.63±0.60 | 8.25±0.78 | 18.70±3.96 |
| 5-*O*-caffeoylquinic acid | 36.76±0.85 | 2.54±0.64 | 4.66±1.20 |
| caftaric acid | 26.53±0.40 | 3.94±0.30 | 10.46±1.27 |

**Table S3** The *in silico* pharmacokinetic parameters of candidate markers.

| No. | GA | Drug-likeness | | | | |
| --- | --- | --- | --- | --- | --- | --- |
|  |  | Lipinski | Ghose | Veber | Egan | Muegge |
| **5** | √ | √ | √ | √ | √ | × |
| **8** | √ | √ | √ | √ | √ | × |
| **18** | √ | √ | √ | √ | √ | √ |
| **19** | √ | √ | √ | √ | √ | √ |
| **20** | √ | √ | √ | √ | √ | √ |
| **21** | √ | √ | √ | √ | √ | √ |
| **1** | × | √ | × | × | × | × |
| **3** | × | √ | × | × | × | × |
| **7** | × | × | √ | × | × | × |
| **9** | × | × | × | × | × | × |
| **13** | × | × | √ | × | × | × |
| **17** | × | × | √ | × | × | × |

**Table S4** Methodology investigation for the quantitation of four markers.

| Compound | Regression equition | r^2^ | Test range (μg/mL) | Repeatability (RSD, %) | Recovery | |
| --- | --- | --- | --- | --- | --- | --- |
|  |  |  |  |  | mean | (RSD, %) |
| cichoric acid | y=39764x-226680 | 0.9994 | 0.44-677 | 1.15 | 100.83 | 2.38 |
| caffeic acid | y=61367x+38743 | 0.9997 | 0.11-166 | 1.95 | 99.68 | 1.05 |
| luteolin | y=42424x+1807.2 | 1 | 0.15-118 | 1.90 | 98.45 | 1.55 |


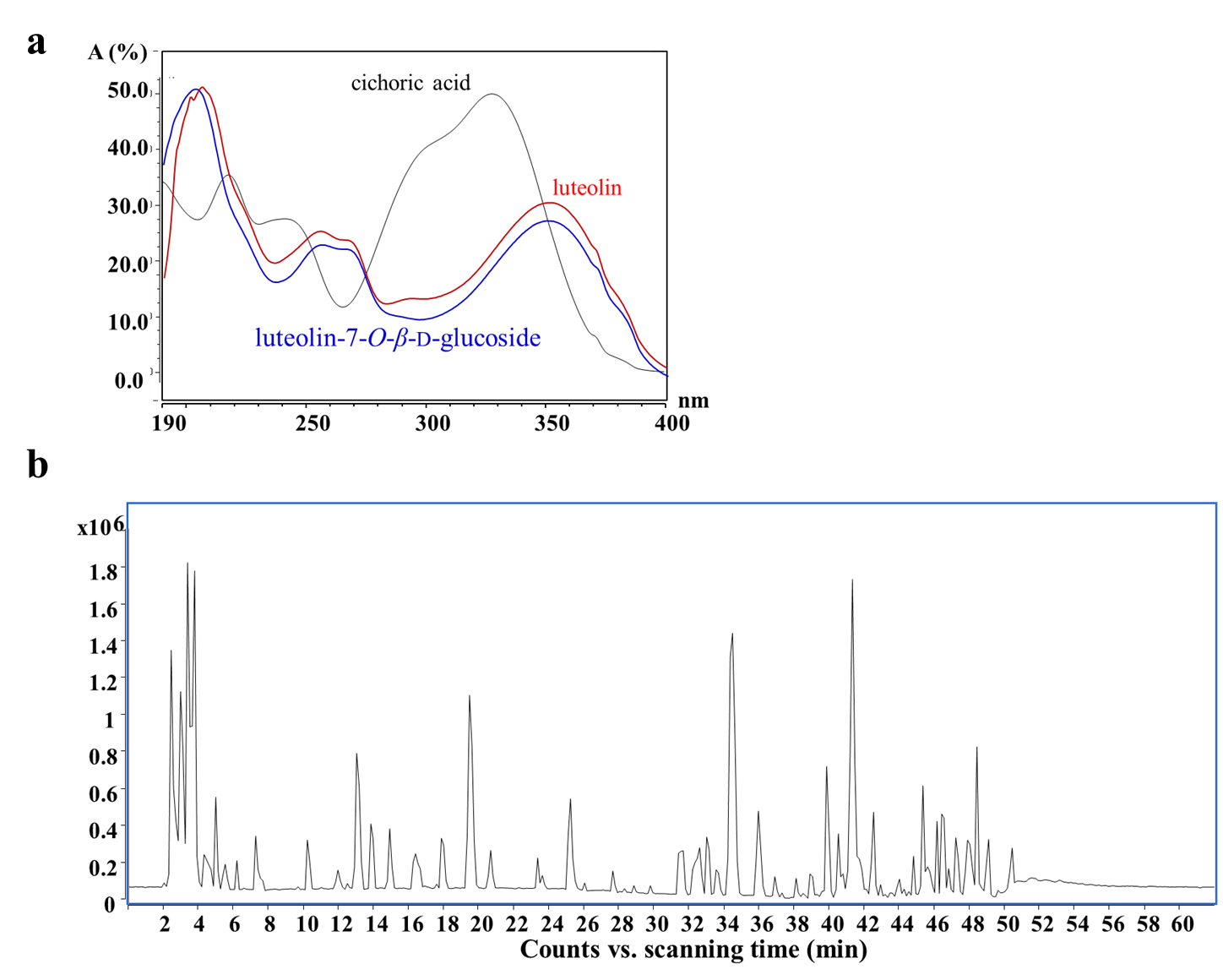


**Fig. S1** Representative UV absorbance profile (**a**), and base peak chromatogram (**b**).


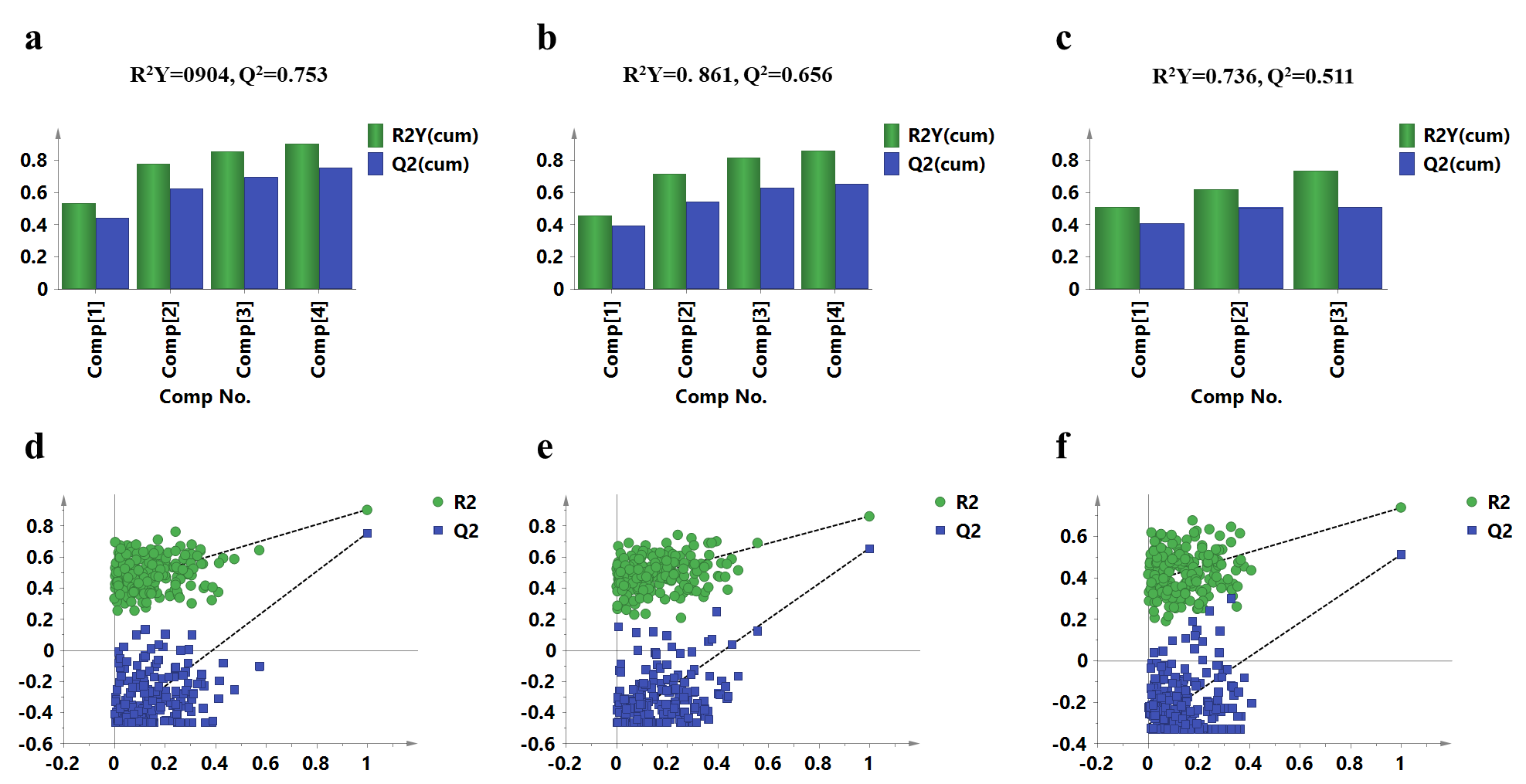


**Fig. S2** PLSR models. The summary of fit (**a-c**) and permutation tests (**d-f**) for antioxidative activity by DPPH and FRAP, and anti-inflammatory activity, respectively.


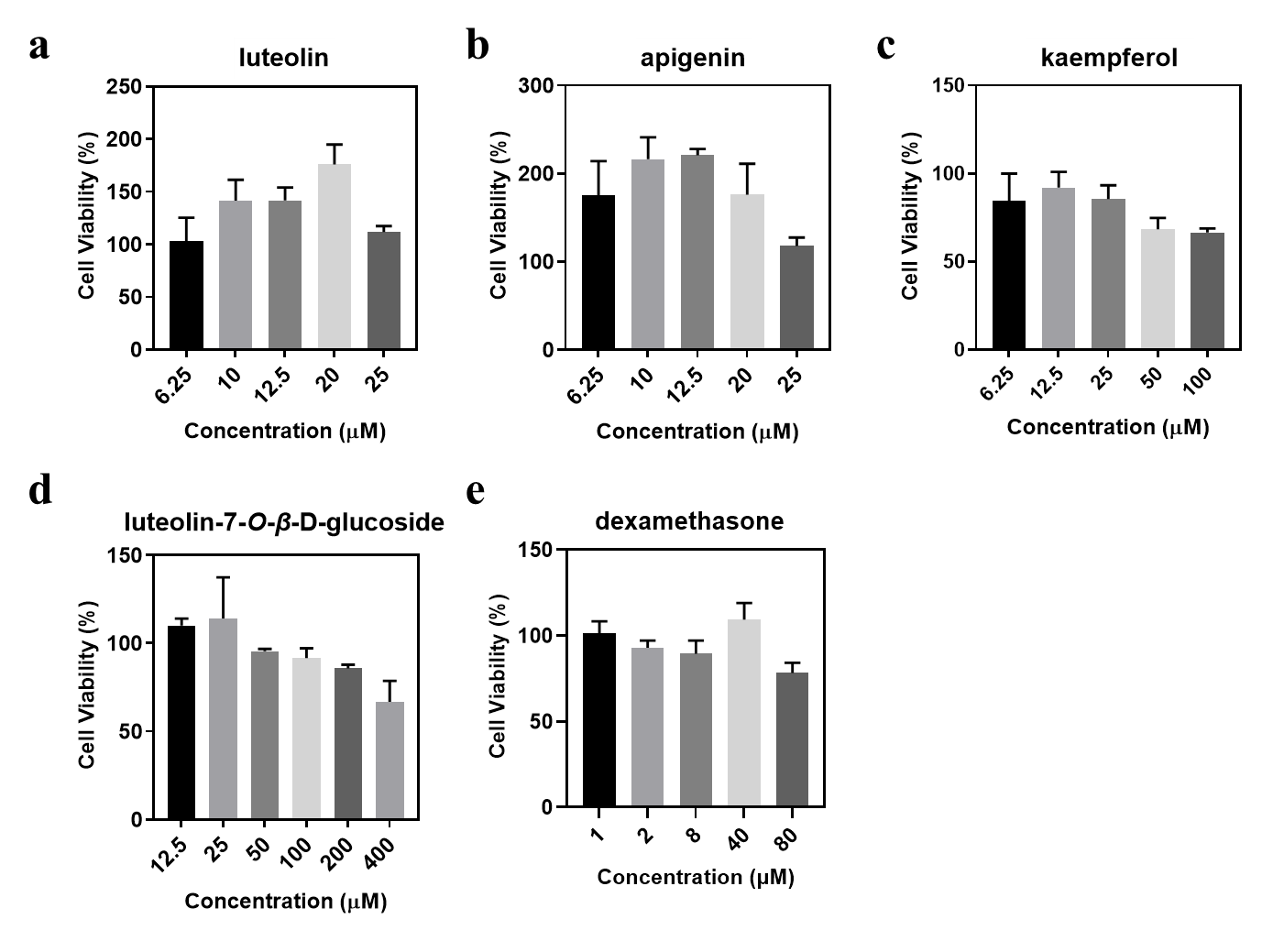


**Fig. S3** The toxicity of luteolin (**a**), apigenin (**b**), kaempferol (**c**), luteolin-7-*O*-*β*-d-glucoside (**d**) and dexamethasone on RAW264.7 cells.

**
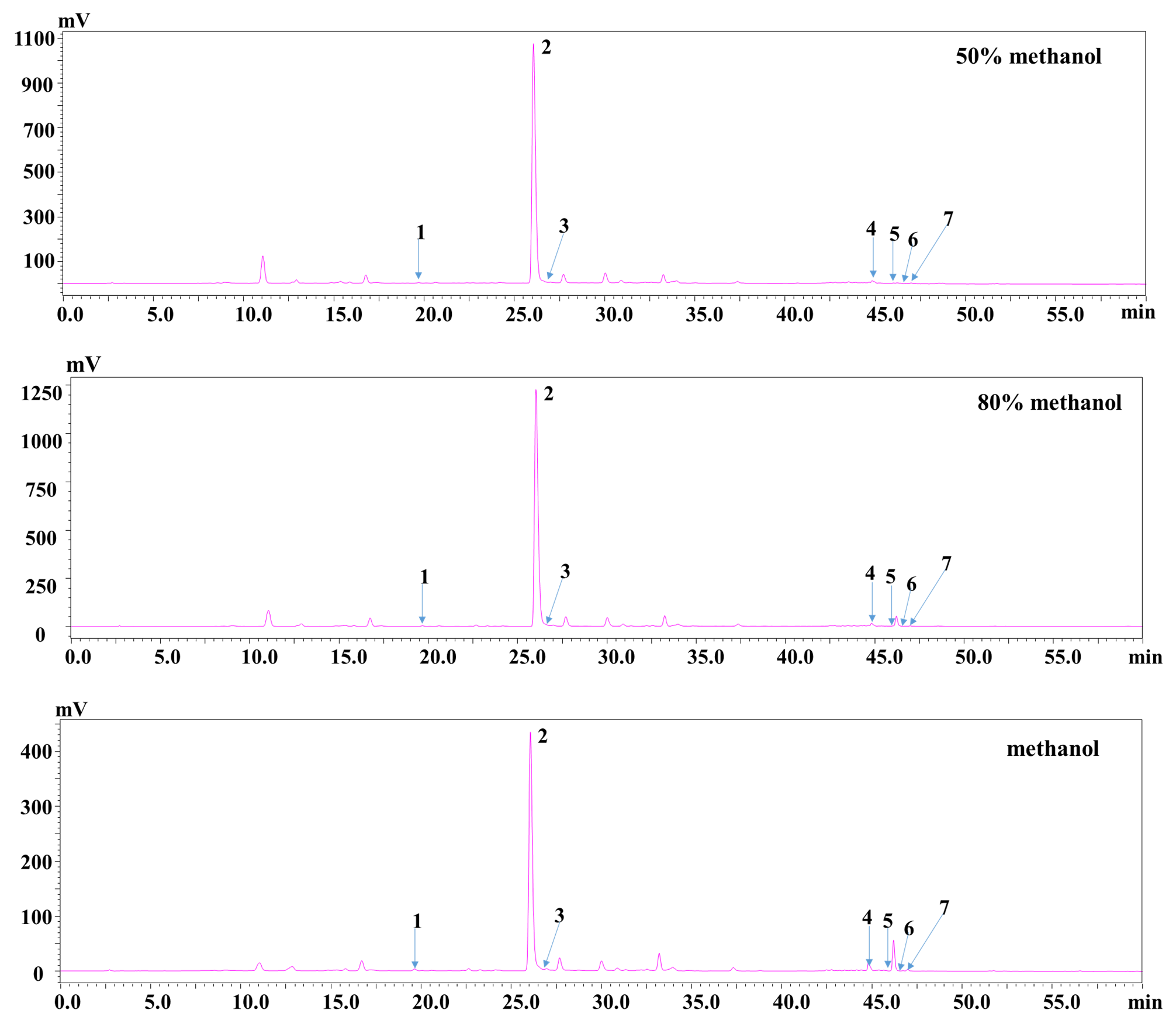
**

**Fig. S4** Chromatograms of TH samples extracted by different solvents. **1** caffeic acid, **2** cichoric acid, **3** *p*-coumaric acid, **4** luteolin, **5** kaemferol, **6** apigenin, **7** eupalitin.
